# What can mathematical modelling contribute to a sociology of quantification?

**DOI:** 10.1057/s41599-023-01704-z

**Published:** 2023-05-06

**Authors:** Andrea Saltelli, Arnald Puy

**Affiliations:** 1 UPF Barcelona School of Management, Barcelona, Spain; 2 Centre for the Study of the Sciences and the Humanities, University of Bergen, Bergen, Norway; 3 School of Geography, Earth and Environmental Sciences, University of Birmingham, Birmingham, UK

**Keywords:** Science, technology and society, Sociology

## Abstract

Sociology of quantification has spent relatively less energies investigating mathematical modelling than it has on other forms of quantification such as statistics, metrics, or algorithms based on artificial intelligence. Here we investigate whether concepts and approaches from mathematical modelling can provide sociology of quantification with nuanced tools to ensure the methodological soundness, normative adequacy and fairness of numbers. We suggest that methodological adequacy can be upheld by techniques in the field of sensitivity analysis, while normative adequacy and fairness are targeted by the different dimensions of sensitivity auditing. We also investigate in which ways modelling can inform other instances of quantification as to promote political agency.

## Introduction

Historians, sociologists and politologists have studied how numbers are produced, used, trusted or feared in relation to different aspects of life, such as empowering systems of governance or control, promoting consumption or consensus, variously facilitating or complexifying human experience. Important contributions to this debate have also come from scholars of law and economics, expressing concern about risks from new numerical technologies and practices. This new attention to numbers, a true sociology of quantification, is an expanding field touching on many families where numbers are produced, from data science to algorithms, quantified self and indicators at various levels of aggregation (Box 1).

Some aspects less visited by these works are the science reproducibility crisis (Saltelli and Funtowicz, 2017; Smaldino and McElreath, 2016) and the important role played by statistics, which has been accused of having permitted misuse or abuse of statistical tests (Leek et al., 2017; Leek and Peng, 2015; Stark and Saltelli, 2018). This omission is all the more surprising as statisticians, mired in the crisis, have been vigorously debating what to do in what have been termed ‘Statistics Wars’ (Amrhein et al., 2019; Mayo, 2018; Wasserstein and Lazar, 2016). Mathematical modelling has been kept out of this debate, partially because it is not a discipline (Saltelli, 2019), and several communities of scientists go about modelling without universally agreed norms of adequacy and quality control (Eker et al., 2018; Padilla et al., 2018).

To complicate matters, the nature of a mathematical model is not easy to define. The Oxford learner dictionary entry mentions “a simple description of a system, used for explaining how something works or for calculating what might happen”. For ecologist Robert Rosen (1991), modelling is not even a science but rather a craft with a central role in science. The extraordinary versatility of models as both instruments to do things and represent phenomena is also noted by Morgan and Morrison (1999). According to these authors, models are partly independent from both the theory and the world they describe, and this versatility enables them to act as mediators in a vast array of tasks. For authors such as Ravetz (2003), models are metaphors and are best used as such—to facilitate dialogue among stakeholders facing a problem that requires mutual understanding. Models are good at mapping one set of meanings or information onto another set, e.g., moving from assumptions to inferences without losing sight, in a correct use, of all the conditionalities involved in this transposition. In a sense, metaphors are themselves models. Adaptive systems have a model of themselves, which allows them to anticipate rather than just adapt (Louie, 2010). According to (Lakoff and Johnson, 2003), we all live and think through metaphors.

Examples of models range from population dynamics to consumer behaviour, from resource management to business accounting, from planetary physics to pricing in finance, from hydrology to university rankings, from optimisation to operational research and so on over a list that is too long to compile in full.

In this paper, we discuss the extent to which two frameworks from mathematical modelling, sensitivity analysis (SA) and sensitivity auditing (SAUD), may be useful to other families of quantification. While SA complements an uncertainty analysis by identifying the inputs/structures that convey the most uncertainty to the model output (Saltelli et al., 2008), SAUD extends the examination to the entire model generation and application process: it aims at unfolding possible stakes, biases, interests, blind spots, overlooked narratives and worldviews of the developers (Lo Piano et al., 2022). Both approaches have the potential to ‘tame’ the opacity of algorithms and apportion the uncertainty and ambiguity of a quantification to its underlying assumptions. Most models in real-life don’t take their input in the form of hard, incontrovertible facts and crisp numbers, but as inputs whose values and meanings are uncertain.

SA and SAUD can check the quality of numbers on the technical and normative dimensions respectively, echoing the double requirement for quantification put forward by Amartya Sen (1990). The idea that the quality of numbers needs technical rigour and normative transparency was at the root of early attempts to advance the use of pedigrees for numbers in policy decisions (van der Sluijs et al., 2005). Both SA and SAUD are inspired by post-normal science (PNS), an approach to science for policy that finds use in the presence of uncertainties, conflicted values and interests, and urgent decisions (Box 2).

The wisdom of SA and SAUD can be translated into a set of precepts to feed into an epistemology of quantification. Since we tend to perceive numbers as more neutral and factual than they actually are, how should we adjust our perception and expectation when a quantification is offered to us? Models and other instances of quantification may come in the form of black boxes or present themselves with considerable interpretative obscurities; we can use here the expression of “hermeneutics of quantification”, as if models were ancient texts whose wisdom must be deciphered.

In the next section, we define uncertainty quantification, SA and SAUD; we then discuss how SA and SAUD can be extended to various instances of quantification using as a starting point recent works for responsible modelling (Saltelli et al., 2020; Saltelli and Di Fiore, 2023). We illustrate how some relevant dimensions of modelling, such as the impossible candour of SA or the concept of modelling of the modelling process, may find their way into sociology of quantification studies. We conclude by examining some policy implications derived from our approach.

Box 1 Studies of quantification The study of quantification is burgeoning with work coming from different fields of scholarship (Di Fiore et al., 2022; Popp Berman and Hirschman, 2018). Two important French schools of sociology of numbers—the so-called Foucauldian studies of quantification and the school of Economics of Convention (Desrosières, 1998; Mennicken and Salais, 2022)—have led to the present movements of “statactivists” under the slogan “another number is possible” (Bruno et al., 2014a). Data scientists, jurists and economists have variously addressed threats coming from different instances of quantification, from misuse of metrics (O’Neil, 2016) to the end of a society ruled by just law (Supiot, 2017) or the advent of surveillance capitalism (Zuboff, 2019). The known seduction of numbers (Merry, 2016), their performativity (Espeland and Sauder, 2016) and their increased penetration in all aspects of life (Couldry and Mejias, 2019) are creating movements of resistance (Algorithmic Justice League, 2020; Bruno et al., 2014a; Cardiff University, 2020) and mediatic echo (Kantayya, 2020, Orlowski, 2020). Anticipated by sociologists of quantification (Espeland and Stevens, 2008), the idea of an “ethics of quantification” to be monitored by societal actors is receiving attention (Saltelli et al., 2021).

Box 2 Post-normal science Post-normal science (PNS) is an approach for the treatment of problems at the science-policy interface (Funtowicz and Ravetz, 1993). PNS applies when problems are characterised by uncertainty, urgency, high stakes and conflicting values. PNS provides epistemological tools to engage with a science that does not pretend neutrality and that aspires to achieve quality rather than universal truth. Many natural scientists increasingly refer to PNS in the treatment of so-called wicked problems (Rittel and Webber, 1973), i.e., problems where the same definition of the issue is contested.

Quantification and mathematical modelling are central to PNS, which critically targets spurious precision, reduction of complexity and transformation of political problems into technical ones via risk or cost-benefit analyses (Funtowicz and Ravetz, 1994). A central concept of PNS is that of a humble science that operates within an *extended peer community*, intended as a community including experts, lay citizens, investigative journalists and whistle blowers—whoever has stakes and interests in the issue being addressed.

### Uncertainty quantification, sensitivity analysis (SA) and sensitivity auditing (SAUD)

Mathematical modelling is not a discipline such as statistics (Saltelli, 2019), so its quality assessment methodologies tend to be scattered among several disciplines (Padilla et al., 2018). Additionally, there are myriads of diverse models and contexts of application. Different taxonomies of models are available as well as several discipline-specific guidelines for model quality.^1^ One of the most relevant acid tests for the quality of models is uncertainty analysis, which quantifies how variable the model-based inference is when the inputs feeding into the model (e.g., parameters, boundary conditions, model structures) are uncertain. This is usually followed by SA to appraise the relative importance that these uncertain input factors have in conveying uncertainty to the model output.

Global SA aims to ensure that the entire space of the input uncertainties is properly explored. The specification ‘global’ is needed here as many SA exercises seen in the literature are ‘local’, i.e., they explore model behaviour only around specific points or axes in the input space and hence do not appraise interactions between inputs (Ferretti et al., 2016). Local methods tend to grossly underestimate the uncertainty in the output because they miss extreme output values produced when all uncertainty inputs are simultaneously varied (Saltelli et al., 2019).

**Fig. 1:**
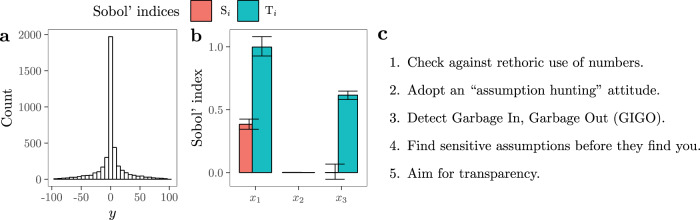
Graphical representation of uncertainty analysis, SA and SAUD. We illustrate the first two approaches using as a toy model the Ishigami and Homma (1990) function, which has three uncertain input factors. **a** Distribution of the model output *y* once uncertainties are propagated through the model. **b** SA of the model output *y*. *S*_*i*_ reflects the first-order effect of the parameter *x*_*i*_, i.e., the proportion of variance conveyed to *y* by *x*_*i*_. *T*_*i*_ denotes the total-order effect of *x*_*i*_, i.e., the first-order effect of *x*_*i*_ plus the effect derived from its interactions with all the other uncertain parameters. Note how the parameter *x*_3_ impacts the model output *y* only through interactions and that *x*_2_ does not convey any uncertainty at all. **c** The five main suggestions of SAUD after Saltelli et al. (2013).

The selection of SA and SAUD as a contribution from mathematical modelling to sociology of quantification appears motivated by these methods’ capacity to probe deep uncertainty (Steinmann et al., 2020), by their visibility in policy-related science (Saltelli et al., 2020), and by their closeness to PNS (Box 3 and Fig. 1).

Box 3 Uncertainty quantification, SA and SAUD Uncertainty analysis: the study of the uncertainty in model output—see also uncertainty cascade (Christie et al., 2011).

SA: the study of the relative importance of different input factors on the model output (Saltelli, 2002).

SAUD: “Sensitivity auditing is a wider consideration of the effect of all types of uncertainty, including structural assumptions embedded in the model, and subjective decisions taken in the framing of the problem” (European Commission, 2021).

## Bridging mathematical modelling with sociology of quantification

In this section, we explore what can SA and SAUD bring to improve the transparency, adequacy and fairness of numbers in quantitative-oriented disciplines, and hence become material for a sociology of quantification. Our discussion draws from the guidelines recently put forward by a work on responsible modelling that merged concepts and approaches from modelling, economy, philosophy and sociology of quantification (Saltelli et al., 2020).

### Mind the assumptions: assess uncertainty and sensitivity

“Sensitivity analysis could help” is the title of a famous article by econometrician Edward E. Leamer (1985), who recommended SA to stress-test econometric studies by changing their modelling assumptions. Another econometrician, Peter Kennedy (2008), made this into one of the commandments of applied econometrics, observing that SA amounts to a sort of confession from the analyst, adding that this confession would ultimately help to anticipate criticism. Note that both Leamer and Kennedy were writing well before the non-reproducibility of large part of economic research became exposed (Ioannidis et al., 2017). In a more recent work, Leamer (2010) commented that the reluctance of modellers to adopt SA is that, in its candour, SA can reveal the fragility of the evidence—“their honesty seems destructive”, adding that “a fanatical commitment to fanciful formal models is often needed to create the appearance of progress.”

While uncertainty can be artificially compressed to defend the relevance of an assessment, it can also be inflated, for example to diminish the relevance of studies conducted by regulators. In the ‘regulation game’^2^ (Owen and Braeutigam, 1978), uncertainty can be played both ways (Oreskes, 2018; Saltelli, 2018) with techniques of increasing sophistication when science and its quantification become functional to processes of regulatory capture (Saltelli et al., 2022).

At the same time, the resistance of some modellers to come to terms with the full uncertainty of their work has motivations such as that of “navigating the political” (defending the role of modelling work in policy relevant settings, see van Beek et al., 2022). This may result in the production of impossibly precise numbers that feed into the policy process. Recent examples are the social cost of carbon, obtained by mathematical simulation of the economy three centuries into the future (Coy, 2021; Rennert et al., 2021), and the unreasonable reliance on an average reproduction rate R for COVID-19 in the course of the pandemic (Miller, 2022). A lucid conclusion reached by philosopher Jerome R. Ravetz is that

We have perhaps reached a complex epistemic state, where on the one hand ‘everybody knows’ that some numbers are pseudo-precise and that numbers can be gamed, while the game works only because most people don’t know about it (Ravetz, 2022).

Sociologist Theodor Porter (2012) noted situations where numbers take centre stage in the public discourse in spite of their fragility. He describes as ‘funny numbers’ those churned out in financial econometrics, one of the causes of the subprime mortgage crisis (Wilmott and Orrell, 2017). Porter points here to the almost comical contrast between the scene where these numbers present themselves with uncontested authority, and the behind-the-scene fights for the interests associated with these numbers.

The construction of a mathematical model often extends in time, with several choices and assumptions implemented during its construction. To achieve a better domestication between models and society we must retrace the steps of the analysis so that influential assumptions having a bearing on the model output are identified and discussed. This modelling of the modelling process can easily be extended to other forms of quantification to reveal the volatility of aggregate or composite indicators (Paruolo et al., 2013; Saisana et al., 2005). The neutrality or ‘facticity’ of a system of indicators can be challenged when different aggregations are compared with one another (Kuc-Czarnecka et al., 2020).

When faced with ambiguities in model formulation the initial instinct of a mathematically trained mind is to fix it, to get the right unambiguous formulation of the problem. In statistics, a very delicate discipline where ambiguity is always behind the corner, this is exemplified by Peter Hand’s (1994) effort at deconstructing and then rectifying poorly posed statistical questions. While this is partly viable for statistics, the messiness of real-life problems where mathematical modelling is applied often prevents such a clear-cut reformulation of context and purpose. This is also because the ambiguity of the problem definition, disliked by mathematical minds, creates in practice the space for negotiation among parties with different cultures, stakes or worldviews. Such idea is behind the concept of “clumsy solutions”:

… solutions are clumsy when those implementing them converge on or accept a common course of action for different reasons or on the basis of unshared epistemological or ethical principles (Rayner, 2012).

By adopting the strategy of modelling of the modelling process one can replace the identification of the right formulation with the exploration of many different formulations. In statistics this has been referred to as the garden of the forking paths (Gelman and Loken, 2013). Based on a short story by Jorge Luis Borges (1941), the garden of the forking paths is a metaphor for the statistician or modeller having to take decision (left or right) in navigating the garden of building a solution to a problem, thus leaving several alternative and potentially legitimate paths unexplored. The solution suggested by SAUD is to take both left and right at each bifurcation and to propagate the uncertainties accordingly.

**Consider the impossible candour of SA and SAUD and the modelling of the modelling process.**

### Mind the hubris: complexity can be the enemy of relevance

Larger models are in general the result of the modellers’ ambition to achieve a better description of their systems and reduce uncertainty through the addition of model detail. There is also a political economy in mathematical modelling whereby larger models command more epistemic authority and better inhibit external scrutiny from non-experts. Such trend towards model complexification leads to overambition and hubris (Puy et al., 2022; Puy et al., 2023; Quade, 1980), two features that also apply to other instances of quantification. For example, composite indicators displaying an impressive number of input variables, meant to convey an impression of complexity and completeness, often depend upon a much smaller subset, suggesting a rhetorical use of numericized evidence.

In modelling, when there are available data against which to compare the model predictions, information criterion such as Akaike’s (2011) or Schwarz’s (1978) can be used to balance model complexity with parsimony. Lacking a validation data set, uncertainty quantification and SA can be used to gauge the uncertainty in the inference and its sources (Puy et al., 2022) For each family of quantification, agreed rules should be established to gauge complexity.

**Consider if the degree of complexity of a quantification can be gauged against agreed criteria.**

### Mind the framing: match purpose and context

Models embed the normative values and worldviews of their designers, and no model can serve all purposes and contexts. They need transparency and non-ritual forms participation (Arnstein, 1969) to realise their potential. Transparency in the frames puts quantification in a context of social discovery (Boulanger, 2014; Dewey, 1938), allowing different frames to be contested and compared as suggested by the French statactivists (Bruno et al., 2014b). The agenda of this movement is to “fight against” as well as “fight with” numbers. Its repertoire of tactics against perceived statistical abuse include:

- Self-defence or “statistical judo”—i.e., gaming the metrics, a strategic use of the Goodhart law.
- Exposing the faults of existing measures, e.g., by denouncing the middle-class bias of the existing French consumer price index (PPI).
- Developing new measures, e.g., a new PPI in defence of the purchasing power of the poor.
- Identifying areas of exclusion and neglect of existing official statistics.

Democracy suffers when numbers are used to create cognitive ambiguity and ensure quantitative justifications that hamper the articulation of alternative legitimate claims (Salais, 2022). Cognitive ambiguity in modelling goes under the name of “displacement”, a term that describes the situation where the attention is focused on the output of a model rather than on the real world purportedly described by the model (Rayner, 2012). Displacement of this nature can be operated via quantification by a plurality of actors, from corporate or political interests to regulators, from issue advocates to the scientists themselves (Saltelli et al., 2022). In the Chesapeake Bay Programme watershed treated by Rayner (2012), the loading of nutrients in the basin is read from the model rather than from the actual basin.

Sociologist of quantification Robert Salais (2022) distinguished *statistics* from *governance-driven quantification*. The former, starting toward the end of the XIX century and lasting well into the XX, was meant to “build things that hold together” (Desrosières, 1998) via a categorisation and classification that created social conventions and concepts to tackle political actions. With *governance-driven quantification*, statistical objects are meant instead to ground (and at the same time foster) policies with preselected objectives. For Salais, this operates a reversal of the statistical pyramid, i.e., starting from the desired political objective to produce the desired system of measurement. In other words, it is a move from evidence-based policy to policy-based evidence aiming at demonstrating that the selected policies are successful.

Quantification thus plays an important role in the context of technocratic approaches to governance (van Zwanenberg, 2020). Some see quantification as a potentially relevant actor in the promotion of inequality and the undermining of democracy, with a combination of (i) the already mentioned “justificationism”, where to objective of a number is to justify a policy, (ii) the pretence of objectivity, whereby the purported neutrality of numbers is used as a shield of facticity against possible ideological resistance (Porter, 1995), and (iii) a tendency to reductionism, whereby complex sociological realities are reduced to simple metrics and the attendant uncertainty is suppressed (Scoones and Stirling, 2020).

For Salais (2022), democracy mutates into a-democracy when the citizens are de facto deprived of agency: they can formally participate, but not influence the outcome of a decisional process. This is where quantification plays an important role by imposing on possible contesters the obligation to articulate alternative claims via an alternative edifice of facts^3^.

The solution to this use of quantification is for Salais the construction of an “Informational Basis of Judgement in Justice” (IBJJ), as proposed by Amartya Sen (1990). For Sen an informational basis should satisfy criteria of fairness, admitting the existence of multiple ‘factual territories’. Adopting Sen’s capability approach, fairness is intended as the freedom for different persons to lead different lives. It is not sufficient for two people to have the same amount of primary goods in order to have the same set of capabilities, as they may differ in their occasion and capacity to transform goods into desired outcomes. For Sen and Salais, technical quality (correctness) for a system of measurement is insufficient if it is not complemented by fairness. The latter can only be achieved if the involved parties have been permitted to negotiate and compromise on what should be measured and how.

**Consider the use of SA and SAUD for the inspection of both technical and normative adequacy.**

### Mind the consequences: quantification may backfire

Models for policy-making that retreat to being “theoretical” or “building blocks” when their unrealistic assumptions are criticised are known as “chameleon models” (Pfleiderer, 2020). This shape-shifting may lead to undesired outcomes, as that of the “funny” numbers of financial econometrics just mentioned (Porter, 2012).

SA and SAUD can contribute to sociology of quantification by deconstructing indicators fraught with important social impact. For example, SA can show how higher education rankings are both fragile (Saisana et al., 2011) and conceptually inconsistent in the way variables are aggregated (Paruolo et al., 2013). This work can support initiatives such as the recent fight against the Word Bank Doing Business Index, closed in 2021^4^. In general, quantitative and qualitative tools developed from SA and SAUD can be used to contrast reductionist or technocratic tendencies on international bureaucracies (van Zwanenberg, 2020), or to broaden the policy definition of an issue. To make an example, many definitions of cohesion (and ways of constructing its indicators) are possible among EU countries, leading to diverging policy implications (Kuc-Czarnecka et al., 2020).

The issue of the perverse effects of algorithms is one of the most visited in sociology and ethics of quantification, as noted above. An interesting line of work suggested by Louise Amoore (2020) concerns the fact that making algorithms “good” or “transparent” is beyond the point. Algorithms create new norms of good or bad, define what is normal and acceptable. Thus, Amoore argues that rather than asking from algorithms an impossible transparency, one should engage with their opacity. To “oppose the violence in the algorithmic foreclosure of alternative futures”, she advocates distributed forms of the writings of algorithms. This would amount to participatory forms of modelling of the modelling process, a programme where the tools suggested here could help.

An interesting application of global SA is in determining a possible incursion of algorithms into revealing “protected attributes” such as gender and race, even if these attributes are not explicitly present in a machine learning algorithms (O’Neil, 2016). A SA of the algorithm’s features can ensure that the algorithm is ‘fair’ in this respect (Bénesse et al., 2021).

**Identify structured strategies to both discuss, negotiate, and or possibly deconstruct measurements, especially in relation to their unintended or malicious effects.**

**Example: use SA to ascertain that an algorithm does not make implicit use of protected attributes.**

### Mind the unknowns: acknowledge ignorance

Often, a political problem is transformed into a technical problem by suppressing uncertainty and using concepts such as “cost-benefit”, “expected utility”, “decision theory”, “life-cycle assessment” or “ecosystem services”, all under the heading of “evidence-based policy” (Scoones and Stirling, 2020; Stirling, 2019). The way modellers can contrast this is by showing that uncertainties are larger than stipulated and by opening the box of quantification to the modelling of the modelling process. Failure to acknowledge ignorance may limit the space of the policy options and offer politicians a way to abdicate responsibility and accountability.

Modellers can also contribute to a sociology of quantification by offering tools to partition the uncertainty in the inference between data-driven and model-driven, or by contrasting prediction uncertainty with policy option uncertainty: if two policy options differ in their outcome by an interval smaller than that governed by data and model uncertainty, then the two options are undistinguishable. For instance, it may be impossible to advocate for incineration or disposal of urban waste when the uncertainty brought about by the system of indicators adopted does not allow to distinguish one option from the other (Saltelli et al., 2000).

SA and SAUD can also be considered as part of the ‘reverse engineering’ operated by data activists in their ‘hackatons’ to bring the normative bias of algorithms to the surface (O’Neil, 2016), as just discussed in relation to protected attributes.

When it comes to methods of quantification, facts and values may be hard to separate. This calls for an integrated assessment of system uncertainties and normative omission or invisibilities. A plain quantitative error propagation analysis (uncertainty quantification) is a valid starting point. It can be used via negativa, i.e., to demonstrate that there is simply not enough evidence to offer a measure, or that the measure is totally driven by untestable hypotheses rather than by available evidence.

**Avoid “quantifying at all costs” and discern when the data available/the scientific goal does not sit well with quantification.**

## Conclusions

Due to the large use of mathematical models during the COVID-19 pandemic of 2020, problematic aspects of mathematical modelling have come to the fore. Models were praised by some for spurring action as well as vilified by others as promoters of ill-conceived policies.

Results from models and other instances of quantification reflect the interests, disciplinary orientations and biases of their promoters, and this has become especially apparent with the pandemic. One cannot but take note of the “dramatic extent to which the people who did best during the pandemic resemble those who built the model” (Winsberg, 2022). Containment measures were evidently more bearable or advantageous for modellers working on their laptop at home than they were for people working at meat-processing plants.

Even the well-meaning quantifier may be tempted to paint bleak futures to prevent them from happening. But this is not what society needs from the use of quantification.

A critical question remains of how we can keep the advantages of encoded mathematics without becoming their victims, or simply subordinates subjects devoid of agency. A related question is how we can do that without being entrapped into the straight jacket of the so-called deficit model, whereby increasing the scientific (model) literacy of citizens would solve our problems. Citizens are not the only subjects whose literacy needs to improve.

Mathematical models, as statistical measures and indicators, can be an important tool of social discovery. Used instrumentally, i.e. to create an illusion of scientificity, models can make this discovery more arduous^5^. Models have thus far remained elusive to tackle for a sociology of quantification: we have statactivists (Bruno et al., 2014a; Samuel, 2022), data activists (Cardiff University, 2020) and a vast movement of sensitisation around the use of algorithms in public matters, e.g., about algorithmic justice (Algorithmic Justice League, 2020). Where are the activists for mathematical modelling?

Following Dewey, the making of democracy is predicated on the existence of publics sharing commonly understood facts. Once the wall of numericized facts is built from above, citizens are cut out from meaningful and deliberative participation. Opposing such a trend needs bridges to be built across all sectors of society. If the “Informational basis of judgement in justice” is where the battle needs to be fought, meaning by this a focus on both the quality of numericized evidence and on its fairness, then an extended peer community involving both modellers and those interested in their use needs to be established. This process may not be entirely peaceful.

## Data Availability

Not applicable as no data were generated or analysed.

## Footnotes

1 Some pointer to literature is available in the supplementary material of Saltelli et al. (2020).

2 “The Regulation Game: Strategic Use of the Administrative Process” is the title of a work by Owen and Braeutigam (1978) that instructs industrial and commercial actors as to how to benefit from administrative and regulatory processes. It argues that regulation can be gamed at the advantage of incumbents, shielding them from competition. The book also instructs lobbyists with remarkable candour as to how they should enrol scientists to defend industrial agenda. This should be done “with a modicum of finesse”, as the expert must now become aware that “they have lost their objectivity and freedom of action” p.7.

3 As an example, Salais compares the concept and indicators of employments as historically determined from the stage of the construction of the concept of employment using statistics to the present period, where the concept of unemployment as a social and statistical category is emptied and is replaced by the maximising of a target of rate of employment. This is achieved by declassifying short periods of unwork (relabelled as transitions) with the result of increased precariousness. In a reductionist move, precariousness is not recognised as a valuable category of social policy (Salais, 2022), p. 388).

4 The index is being reconsidered at the moment of writing the present work as Business Enabling Environment (BEE, Cobham, 2022), an indication of the high stakes associate with this measure.

5 When pragmatist philosopher John Dewey discussed the concept of social discovery in the 30’s he noted that there are ‘publics’ affected by transaction taking place somewhere else. “[…] machine age has so enormously expanded, multiplied, intensified and complicated the scope of the indirect consequences […] that the resultant public cannot identify and distinguish itself” (Dewey, 1938). Dewey’s warning takes on a new urgency now that the machine age has expanded to artificial intelligence and new media, colonising hitherto virgin aspects of human existence (Couldry and Mejias, 2019; Lanier, 2006; Zuboff 2019). Models are part of this picture, potentially helpful or harmful, as a particularly effective instruments for the displacement of attention (Rayner, 2012).
